# The Impact of Different Weed Management Systems on Weed Flora and Dry Biomass Production of Barley Grown under Various Barley-Based Cropping Systems

**DOI:** 10.3390/plants11060718

**Published:** 2022-03-08

**Authors:** Muhammad Naeem, Shahid Farooq, Mubshar Hussain

**Affiliations:** 1 Department of Agronomy, Bahauddin Zakariya University, Multan 60800, Pakistan; naeem_agrarian@yahoo.com; 2 Department of Plant Protection, Faculty of Agriculture, Harran University, Şanlıurfa 63250, Turkey; csfa2006@gmail.com; 3 School of Veterinary and Life Sciences, Murdoch University, 90 South Street, Murdoch, WA 6150, Australia

**Keywords:** weeds, allelopathy, barley, false seedbed, cropping system

## Abstract

Weeds are among the major issues responsible for reduction in yield and profit in any crop production system. Herbicides are the easiest and quickest solution of weeds; however, their frequent use exert negative consequences on environment, human health, and results in the evolution of herbicide-resistant weed species. Due to these reasons, alternative weed management methods that are less harmful to environment and human health are needed. This two-year study evaluated the impact of different weed management options, i.e., false seedbed (FS), allelopathic water extracts (AWE), chemical control (CC), weed-free (WF) weedy-check (WC) on weed spectrum in various barley-based cropping systems, i.e., fallow-barley (FB), maize-barley (MB), cotton-barley (CB), mungbean-barley (M*B), and sorghum-barley (SB). Data relating to density, diversity, and biomass production of weed species prevailing in the studied cropping systems were recorded. Interactive effect of weed management methods and barley-based cropping systems significantly altered weed diversity, and densities of individual, broadleaved, and grassy weeds. A total 13 weed species (ten broadleaved and three grass) were recorded during both years of study. The highest dry biomass, diversity, and density of individual, broadleaved, and grassy weeds were noted in WC treatment, whereas WF treatment resulted in the lowest values of these traits. Chemical control resulted in the highest suppression of weed flora and improved dry biomass production of barley followed by AWE. The SB cropping system with CC or AWE resulted in the least weed flora. The M*B cropping system with CC or AWE produced the highest dry biomass of barley. It is concluded that including sorghum crop in rotation and applying AWE could suppress weeds comparable to herbicides. Similarly, including mungbean in rotation and applying AWE could increase dry biomass production of barley. In conclusion, herbicides can be replaced with an eco-friendly approach, i.e., allelopathy and inclusion of sorghum crop could be helpful in suppressing weed flora.

## 1. Introduction

Barley (*Hordeum vulgare*) is the fourth major cereal in terms of production globally after wheat, maize, and rice. Barley is grown for fodder, brewing, human food, and in the production of malt around the world [1,2]. Barley is cultivated in ~100 different countries [3]. It performs better in low rainfall areas where other crops fail to establish and can survive under adverse environmental and conditions [4,5]. However, it gives better production on moderately saline soils and higher salinity could obstruct its growth leading to reduced yield [6]. Barley is tolerant to several abiotic and biotic conditions; nonetheless, weed infestation can significantly reduce its yield and productivity [7,8,9].

Weeds exert negative impacts on quality and quantity of agricultural products; thus, reduce farmers’ profits to a significant extent [10]. Weeds compete with crop plants either through competing for moisture, sunlight, nutrients, and space or through secreting allelochemicals, which adversely impact seed germination and growth of crop plants [11,12,13]. Nevertheless, weeds produce significant number of seeds, which are deposited to soil seed bank; thus, laying the foundation for future weed infestation [14,15,16]. Therefore, weeds must be controlled to reduce weed seed bank in soil and crop yield losses [17,18]. Several weed management methods, i.e., cultural, chemical, mechanical, and biological are opted to suppress the growth of weed flora [19,20]. Labor unavailability and high wages along with unreliable weed control are the main issues faced in manual/cultural weed control [21]. Mechanical weed control, on the other hand, is expensive because of sophisticated equipment required for each crop [22], and involves extra soil disturbance resulting in the disruption of soil structure and reduced soil fertility [23]. Similarly, frequent use of herbicide in chemical weed control results in the evolution of herbicide-resistant weeds, environmental contamination, and health hazards [20,24].

Because of the disadvantages associated with the prevalent weed management methods, alternative weed control methods with low environmental contamination, health hazards, and lesser herbicide resistance are needed [24]. Adoption of preventive weed control method like false or stale seedbeds provide effective weed control during crop growth with less labor cost [25,26,27]. However, the efficacy of such methods is strongly reliant on time available for the preparation of stale seedbeds, method used, and soil and climatic conditions [25,26]. Recently, plant-based natural products that could serve as alternatives to herbicides have been focused on weed management research globally [28]. Residues’ incorporation of allelopathic crops, and inclusion of such crops in rotation could improve weed control [8,11,29]. The crops with high allelopathic potential include sunflower, rye, wheat, rice, barley, and sorghum, which have been shown to suppress weed flora in different crops [29,30,31]. The allelopathic compounds found in mulberry (tannins steroids and phenols), sunflower (phenolic compounds and terpenes), and sorghum (sorgoleone and phenolics) are responsible for suppression of weed flora [13,29,32,33,34].

Diversifying the crops to be sown on a particular area could suppress weed flora since it has the potential to inhibit weed growth [35,36]. Selection of similar crops for longer time periods results in the proliferation and establishment of particular weed species, which become established and are difficult to control [37]. The inclusion of allelopathic crops, i.e., sorghum in rotation could provide significant control over weeds compared to a rotation having no allelopathic crop [34]. Sorghum releases various allelopathic compounds from its grains, stems, and root hairs; thus, considered as an important candidate for crop rotation to suppress weed flora [30,34]. Several studies explored the allelopathic potential of sorghum as cover crop, mulch, and aqueous extracts on different weeds and concentration-dependent, selective, and species-specific allelopathic effects have been reported [7,8,20,38,39]. Therefore, inclusion of sorghum in barley-based cropping system could suppress weed flora.

This two-year field experiment evaluated the effect of different weed control methods on weed infestation and dry matter production of barley in different barley-based cropping systems. It was hypothesized that different weed control methods will differ in weed infestation level, density, and composition of weed flora. It was further hypothesized that barley-based cropping systems including an allelopathic crop would have lower weed infestation compared to those having no allelopathic crop. The results would help to improve the weed control in barley-based cropping systems and lower the adverse impacts of herbicides on environment and human health.

## 2. Results

### 2.1. Weed Flora

A total 13 weed species (ten broadleaved and three grass) were recorded from the study area during both years of the study. Of the recorded weed species, four were perennial, whereas the remaining nine had an annual life cycle. The weed species belonged to 11 pant families, of which Asteraceae and Leguminosae were represented by two species each, while the remaining families were represented by one species only (Table 1).

### 2.2. Weeds Diversity (Number of Weed Species)

**Figure 1:**
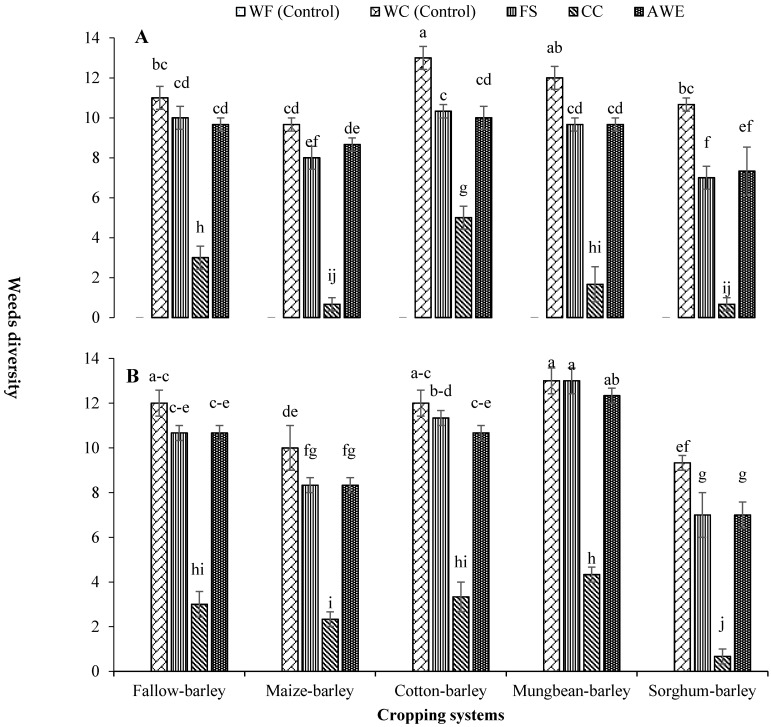
Influence of different barley-based cropping systems on weed diversity (number of weeds species) under various weed management methods during 2017–2018 (**A**) and 2018–2019 (**B**) ±S.E. In the legend, WF = weed free (control), WC = weedy check (control), FS = false seedbed, CC = chemical control, AWE = allelopathic water extracts. The means sharing the same letters do not differ significantly at *p* ≤ 0.05 (LSD value at *p* ≤ 0.05 for 2017–2018 = 1.41, 2018–2019 = 1.37).

The interaction between barley-based cropping systems and weed control methods (WCM) significantly altered weeds’ diversity during both years (Figure 1). During the first year of study, the highest weed diversity was recorded in cotton-barley (CB) cropping system with weedy-check (WC) treatment, which was like mungbean-barley (M*B) system with WC condition. During the second year, M*B cropping system with WC and false seedbed (FS) treatments resulted in the highest weeds’ diversity, which was similar to M*B system with allelopathic water extracts (AWE), and CB and fallow-barley (FB) systems with WC treatment (Figure 1). However, all cropping systems with chemical control (CC) during the first year and sorghum-barley (SB) cropping system with CC during the second year observed the lowest weed diversity (Figure 1).

### 2.3. Density of Broadleaved Weed Species

Barley-based cropping systems, WCM, and their interaction had significant effect on the density of broadleaved weed species. The highest and the lowest density of broadleaved weed species was noted for WC and weed-free (WF) treatments, respectively (Table 2). Chemical control resulted in higher reduction in the density of broadleaved weed species compared to FS and AWE during both years of study. The CB and M*B cropping systems observed the highest density of broadleaved weed species during the first and second years, respectively, while the lowest density of broadleaved weed species was recorded for SB cropping system during both years (Table 2). Regarding interaction, the highest density of broadleaved weeds was recorded in CB cropping system during the first year and M*B cropping system during the second year with WC treatment, whereas all cropping systems (CS) with WF and CC treatments had little or no infestation of broadleaved weed species during both years (Table 2).

### 2.4. Density of Grassy Weed Species

The individual and interactive effects of barley-based cropping systems and WCM had significant effect on the density of grassy weed species (Table 2). The highest and the lowest density of grassy weed species was noted for WC and CC treatments, during both years (Table 2). Regarding CS, FB cropping system had the highest and SB as well as maize-barley (MB) cropping systems recorded the lowest density of grassy weed species density during the first year. However, FB and M*B systems resulted in the highest density of grassy weed species, whereas SB system had the lowest density of grassy weed species during the second year (Table 2). Regarding interaction, FB cropping system with WC treatment had the highest density of grassy weeds, while SB and MB cropping systems with CC recorded the lowest density of grassy weed species during the first year of the study. Similarly, FB, CB, and M*B cropping systems with WC treatment recorded the highest density of grassy weed species, whereas SB cropping system with CC resulted in the lowest density of grassy weed species during the second year of the study (Table 2).

### 2.5. Density of Individual Weed Species

Different barley-based cropping systems, WCM and their interaction significantly altered density of individual weed species during both years (Table 3, Table 4, Table 5 and Table 6). The highest and the lowest density of salt marsh (*Bolboschoenus maritimus* (L.) Palla) was recorded for WC and WF treatments, respectively, during both years (Table 4). The highest density of salt marsh was noted for FB cropping system during the first year, whereas the lowest density was recorded for SB and MB cropping systems during both years (Table 3). Regarding interaction, FB cropping system during 1st year and M*B cropping system during the second year with WC treatment had the highest density of salt marsh, whereas the lowest density was recorded in all cropping systems with CC treatment during both years (Table 3).

The highest density of corn spurry (*Spergula arvensis* L.) was found in WC treatment, while the lowest density was noted for CC and WF treatments during both years (Table 3). The CB cropping system recorded the highest corn spurry density, while the lowest was recorded for SB and MB cropping systems during the first year (Table 3). However, FB cropping system noted the highest corn spurry density during the second year, while no infestation was noted in SB system during the second year (Table 3). Regarding interaction, CB cropping system with WC treatment observed the highest density of corn spurry during the first year. Similarly, the highest corn spurry density was noted in FB cropping system with WC treatment during the second year. However, there was little, or no infestation recorded for all cropping systems with CC and WF treatments during the second year (Table 3).

Winter grass (*Polypogon monspeliensis* L. Desf.) was only recorded during the first year, while no infestation of this weed was noted during the second year (Table 3). The WC treatment had the highest density of winter grass, while CC and AWE treatments had the lowest infestation like WF treatment (Table 3). The CB system recorded the highest density of winter grass, while no infestation of this weed was recorded in MB and SB cropping systems. Regarding interaction, the highest density of winter grass was observed in CB cropping system with WC treatment, whereas all cropping systems with CC and AWE had low or no infestation like WF treatment (Table 3).

The highest common goosefoot (*Chenopodium album* L.) density was noted for WC treatment, while the lowest infestation was recorded for CC and WF treatments during both years (Table 4). The FB, CB, and M*B cropping systems recorded the highest, while MB and SB cropping systems had the lowest common goosefoot infestation during the first year (Table 4). However, M*B cropping system recorded the highest, while SB and MB cropping systems observed the lowest common goosefoot density during the second year (Table 4). Regarding interaction, M*B cropping system with WC treatment had the highest common goosefoot density, while all cropping systems with CC and WF treatments recorded no infestation of this weed during both years (Table 4).

Weedy-check treatment recorded the highest perennial sow thistle (*Sonchus arvensis* L.) density, while the lowest density was noted in CC and WF treatments during both years (Table 4). Cropping systems had non-significant effect on perennial sow thistle density during both years (Table 4). Regarding interaction, the highest perennial sow thistle infestation was noted in M*B and FB cropping systems with WC treatment during the first year. The M*B cropping system with WC treatment recorded the highest infestation of perennial sow thistle, which was statistically at par with CB cropping system under WC treatment during the second year (Table 4). However, all cropping systems with CC and WF treatments observed little or no infestation of this weed during both years (Table 4).

The highest bitter dock (*Rumex obtusifolius* L.) density was recorded in WC treatment, while no infestation of this weed was found in CC and WF treatments during both years (Table 4). The CB cropping system during the first year and M*B as well as FB cropping systems during the second year recorded the highest bitter dock infestation, while the lowest infestation was noted in SB cropping system during both years (Table 4). Regarding interaction, the highest density of bitter dock was noted in CB and M*B cropping systems with WC treatments during the first and second year, respectively. No infestation of this weed was noted in all cropping systems with CC and WF treatments during both years (Table 4).

Weedy-check treatment had the highest and CC as well as WF treatments recorded the lowest fat hen (*Chenopodium murale* L.) density during both years (Table 4). The FB cropping system recorded the highest density of fat hen, while the lowest density was recorded for SB and MB cropping systems during both years (Table 4). The FB cropping system with WC resulted in the highest fat hen infestation, while the lowest infestation was noted in all cropping system with CC and WF treatments during both years (Table 4).

The highest density of field bindweed (*Convolvulus arvensis* L.) was recorded for WC treatment, whereas the lowest was noted for CC and WF treatments during the first year. However, all WCM except FS recorded no infestation during the second year (Table 5). All cropping systems had non-significant effect on field bindweed density during both years (Table 5). Regarding interaction, the highest infestation of field bindweed was recorded in M*B cropping system with WC treatment, while little or no infestation was recorded in all cropping systems with CC and WF treatments (Table 5).

Weedy-check treatment resulted in the highest density of yellow trefoil (*Medicago polymorpha* L.), while the lowest density was recorded for CC and WF treatments during both years (Table 5). The FB, CB, and M*B cropping systems observed the highest infestation of yellow trefoil density, while the lowest infestation was recorded in SB cropping system during the first year (Table 5). Nonetheless, the highest density of yellow trefoil was noted in M*B and FB cropping systems, while MB and SB cropping systems recorded the lowest density during the second year (Table 5). The M*B cropping system with WC treatment had the highest infestation of yellow trefoil during the first year. During the second year, M*B and FB cropping systems with WC treatment observed the highest density of yellow trefoil (Table 5). The lowest infestation of yellow trefoil was observed in all cropping systems with CC and WF treatments during both years (Table 5).

The highest and the lowest density of yellow sweet clover (*Melilotus indicus* (L.) All.) was recorded for WC treatment, and CC and WF treatments, respectively, during both years (Table 5). The CB cropping system had the highest and SB cropping system recorded the lowest density of yellow sweet clover during the first year. The M*B and FB cropping systems noted the highest, while SB and MB cropping systems recorded the lowest infestation of yellow sweet clover during the second year (Table 5). The CB cropping system with WC treatment had the highest yellow sweet clover density during the first year; however, M*B cropping system with WC treatment had the highest yellow sweet clover density during the second year. The lowest density of yellow sweet clover was noted in all cropping systems under CC and WF treatments during both years (Table 5).

Weedy-check treatment had the highest infestation of swine cress (*Coronopus didymus* L. Sm.), while CC, AWE, and WF treatments recorded no infestation during the first year (Table 5). During the second year, WC treatment observed the highest swine cress density, while CC and WF treatments recorded no infestation (Table 5). Cropping systems had non-significant effect on swine cress density during the first year; however, M*B and CB cropping systems observed the highest, while SB and MB cropping systems resulted in the lowest infestation of swine cress during the second year (Table 5). Regarding interaction, FB cropping system with WC treatment resulted in the highest swine cress density, while no infestation was noted in all cropping systems with CC, FS, and WF treatments (Table 5). The M*B cropping system with WC treatment recorded the highest swine cress density, while no infestation was noted in all cropping systems under CC and WF treatments during the second year (Table 5).

All cropping systems with all WCM recorded no infestation of blue pimpernel (*Anagallis arvensis* L.) during the first year (Table 6). However, WC treatment had the highest, while CC and WF treatments recorded no infestation during the second year (Table 6).

No horseweed (*Conyza stricta* Willd.) infestation was recorded in all cropping systems under all WCM during the first year (Table 6). However, WC treatment observed the highest horseweed density, while CC, FS, and WF treatments recorded no infestation during the second year (Table 6). There was non-significant effect of cropping systems during the second year (Table 6). Regarding interaction, M*B cropping system with WC treatment recorded the highest horseweed density, while all cropping systems under CC, FS, and WF treatments, all cropping systems except M*B system under AWE, MB, and SB cropping systems under WC treatment did not have any of this weed during the second year (Table 6).

Dry biomass yield of barley was significantly (*p* < 0.05) influenced by WCM and CS; however, their interactive effect was non-significant during both years (Table 7). The FB cropping system had the lowest, whereas M*B system recorded the highest dry biomass yield, which was at par with MB cropping systems during the first year. However, FB system recorded the lowest dry biomass yield, which was statistically similar to SB system, while the highest was recorded in M*B system during the second year (Table 7). In case of WCM, WF treatment produced the highest dry biomass yield of barley, which was at par with CC treatment, while the lowest was recorded in FS treatment, which was similar to AWE treatment during the first year of the experiment. Nonetheless, WF treatment recorded the highest, and FS, as well as AWE treatment, had the lowest dry biomass yield during the second year (Table 7).

## 3. Discussion

Weed flora, including weed diversity and densities of broadleaved, grassy, and individual weed species were significantly altered by different barley-based CS, WCM, and their interaction (Figure 1, Table 1, Table 2, Table 3, Table 4, Table 5 and Table 6). This supported our hypothesis that barley-based cropping systems and WCM would differ for weed flora and dry biomass production of barley. The highest density and diversity were recorded in WC treatment, while the lowest were noted in WF treatment. The weeds were efficiently controlled by CC as compared to the rest of WCM used in the study. Interestingly, AWE provided sufficient control over weeds after CC indicating that this method could be used to mitigate the adverse effects associated with CC. The highest weeds’ diversity and density were recorded in CB and M*B cropping systems during the first and second year, respectively. The lowest was noted in SB cropping system (Figure 1, Table 2, Table 3, Table 4, Table 5 and Table 6).

Weed diversity and density were significantly reduced by herbicides compared to weedy-check treatment in wheat crop [20]. However, unnecessary and overuse of herbicides has increased the evolution of herbicide resistance in several weed species [40]. Similarly, widespread use of herbicides causes anxiety in the public regarding the adverse effects on the environment and human health [41]. Therefore, it is essential to use some integrated weed management approaches for efficient weed control [42]. False or stale seedbed is regarded as an efficient integrated weed management method as it significantly reduced weeds density and dry biomass compared to WC [20,25,26]. Some weeds like *Eclipta prostrata* L., *Fimbristylis miliacea* (L.) Vahl, *Cyperus iria* L., *Leptochloa chinensis* (L.) Nees, and *Cyperus difformis* L. are comparatively more influenced by FS treatment due to their inability to emerge from a depth >1 cm and low seed dormancy [43]. In this experiment, weed density and diversity were significantly reduced by AWE due to phytotoxic effects. However, FS failed to suppress weed flora. The possible reasons of FS failure are unavailability of sufficient moisture before true seedbed, which reduced seed germination of weed species. Allelopathic water extracts inhibit photosynthesis, cell division, thickness of seminal roots, protein synthesis, and respiration by reducing nutrients and water uptake through roots, which negatively affect weed growth [44]. Sorghum is a renowned allelopathic crop that has the ability to control weed growth owing to the release of sorgoleone from roots [34]. Members of the Brassicaceae family release glucosinolate that gets decomposed into many biologically active compounds, including isothiocyanate [45]. Isothiocyanate effectively suppresses weed growth [46]. Weeds are also controlled by the allelochemicals of sunflower (terpenes and phenolic compounds) [32] and mulberry (steroids, phenols, and tannins) [33]. Therefore, weeds could be controlled by the combination of sorghum, sunflower, eucalyptus, and mulberry alleopathic water extracts.

The CB and M*B cropping systems had the highest weed diversity and density, while SB cropping system recorded the lowest in this regard (Figure 1, Table 2, Table 3, Table 4, Table 5 and Table 6). Similar results were reported by Shehzad et al. [20], where fallow-wheat and rice-wheat rotations favored different weed flora, while sorghum–wheat rotation reduced weed growth. In the current study, CB and M*B cropping systems favored the infestation of common goosefoot, bitter dock, yellow trefoil, yellow sweet clover, and swine cress. Similarly, FB cropping system observed the highest density of yellow sweet clover, yellow trefoil, fat hen, bitter dock, common goosefoot, corn spurry, and salt marsh, while SB cropping system recorded the lowest weed density. Rotating crops that have different cultivation practices or life cycles is an efficient cultural practice for controlling problematic weed species through disturbing their life cycles [47]. It is an effective approach to control weeds; however, it is more efficient when combined with any other weed management practice [48]. Similarly, weeds are suppressed by different management method and the inclusion allelopathic crops in rotation [49]. Different experiments showed that the growth of cultivated crops is significantly affected by allelochemicals exuded from sorghum roots [13,49]. Therefore, the lowest weeds population was recorded in SB cropping system during both years in the current study.

The highest dry biomass yield of barley was noted in WF treatment, while the lowest was recorded in WC treatment (Table 7). The M*B cropping system had the highest dry biomass yield, while the lowest was recorded in FB cropping system (Table 7). The FB cropping system had more weed infestation, which reduced yield-related traits of barley [8]. Weeds negatively affect crop growth by competing for nutrients and other essential resources [50]. However, crops can perform better in the absence of weeds [20].

The M*B cropping system improved dry biomass yield due to better soil condition resulting in better allometric traits and root growth. Therefore, plants dry biomass yield was improved by absorbing more water and nutrients from soil. It has been described by Zhao et al. [51] that the soil fertility and crop productivity can be efficiently increased by practicing legume-based crop rotation. Crop diversification with legumes had significant effect on soil fertility as it improves the status of phosphorus nitrogen, carbon, and soil organic carbon depending upon the soil type [52]. Similar results were reported in the current study.

## 4. Materials and Methods

### 4.1. Experimental Site and Soil

This experiment was conducted during 2017–2018 and 2018–2019 at the Agronomic Research Area, Department of Agronomy, Bahauddin Zakariya University, Multan (30.2° N, 71.43° E and 122 m above sea level), Pakistan. The study area had an arid to semi-arid climate. Weather data of the experimental site during study period are given in Table 8. The study site has loamy soil with pH values of 8.20–8.25, ECe 2.78–2.80 mS cm^−1^, 0.60–0.63% organic matter content, 0.03% total nitrogen, 7.25–7.18 mg kg^−1^ available phosphorus and 240–230 mg kg^−1^ available potash during the first and second year of the study, respectively.

### 4.2. Experiment Description

Barley was cultivated in five different cropping systems, i.e., fallow-barley (FB), maize-barley (MB), cotton-barley (CB), mungbean-barley (M*B), and sorghum-barley (SB). Similarly, five different weed control methods, i.e., weed-free (control; WF), weedy-check (control; WC), false seedbeds (FS), chemical control (CC), and allelopathic water extracts (AWE) were used to test their impact on weed flora and biomass production of weeds and barley. Regarding WF treatment, the weeds were completely removed from the experimental plots during the entire growth period of barley crop, whereas weeds were retained for the whole cropping period in WC treatment. In case of FS treatment, experimental field was tilled and kept fallow for seven days to allow weeds’ growth. Afterwards, the emerged weeds were removed by cultivating the field and seedbed was prepared. For CC treatment, ‘Bromoxynil + MCPA’ (60% EC) was sprayed @1.25 L ha^−1^ after one week of 1st irrigation. In AWE treatment, water extracts of mulberry, sorghum, eucalyptus, and sunflower were prepared and mixed in equal ratio. Afterwards these were sprayed @12 L ha^−1^ after one week of 1st irrigation. The leaves and branches of all crops were taken, chopped into small pieces, and dried under sun for the preparation of AWE. The dried materials were then soaked in distilled water (1:20 ratio), separately for 24 h. The solutions were filtered after 24 h to obtain the extracts. The resulting extracts were then mixed in a 1:1:1:1 ratio, diluted by 10 times, and sprayed. Each treatment was replicated three times and net subplot size was 2.7 × 5 m. The study was carried out according to randomized complete block design (RCBD) with factorial arrangement. Barley-based cropping systems were the main factor, whereas weed control methods were considered as a sub-factor.

### 4.3. Crop Husbandry

Before sowing of all crops, 10 cm irrigation was applied to whole field during both years of study. Afterwards, seedbeds of all crops were prepared once the soil attained feasible moisture level. All crops were sown according to their recommended production technology as given in Table 9. All crops were irrigated by surface irrigation method to fulfill their moisture requirements. All agronomic and plant protection measures were adopted to ensure healthy crop and to avoid pest and diseases. Finally, all crops were harvested at their harvest maturity.

### 4.4. Weeds Data Collection

Data relating to weeds’ diversity (number of weed species), density of broad-leaved and grassy weeds, and density of all individual weeds were recorded at 60 DAS during both years of study. Data were collected from three randomly selected locations in each experimental plot with the help of 1 m^2^ quadrate [8,37]. Weed diversity was recorded by observing all species in 1 m^2^ at three random places in each experimental unit and averaged. Total number of weed species present in each quadrate were noted, identified, and averaged to record the weeds’ diversity. The densities of broadleaved, grassy, and individual weeds were recorded by randomly placing the quadrate at three different places in each experimental unit. The observed weed species for density were separated into broadleaved, grassy, and individual weeds.

### 4.5. Biomass Yield

Two central rows of barley from each experimental unit were harvested at 105 DAS. The barley plants were manually harvested at ground level to observe biomass production. The harvested samples were sun-dried for three days and then oven-dried at 75 °C until constant weight. Dry weight of these samples was recorded by using a digital weighing balance.

### 4.6. Statistical Analysis

The data were tested for differences among experimental years, which indicated that years’ effect was significant. Therefore, data of each year were analyzed and interpreted separately. Collected data for both years statistically analyzed by analysis of variance (ANOVA) [53] according to general linear model procedure. Treatments means were compared by least significance difference (LSD) test at 5% probability level, where ANOVA indicated significant differences.

## 5. Conclusions

Different barley-based cropping systems and weed control methods significantly altered weed flora during both years of the current study. Chemical control resulted in the highest suppression of weed flora and improved dry biomass production of barley followed by allelopathic crop water extracts. The SB cropping system with chemical control or allelopathic crop water extracts resulted in the lowest weed infestation. The M*B cropping system with chemical control, or allelopathic crop water extracts produced the highest dry biomass of barley. It is concluded that including sorghum crop in rotation and applying allelopathic extracts could suppress weeds comparable to herbicides. Similarly, including mungbean in rotation and applying allelopathic extracts could increase dry biomass production of barley. In conclusion, herbicides can be replaced with an eco-friendly approach, i.e., allelopathy and inclusion of sorghum crop could be helpful in suppressing weed flora.

## Figures and Tables

**Table 1:** Common and Latin names, family, and life cycle of different weed species recorded in barley crop during both years of the study.

Species	Common Name	Family	Life Cycle
Broadleaved weed species			
*Chenopodium murale* L.	Fat hen	Amaranthaceae	Annual
*Melilotus indicus* (L.) All.	Yellow sweet clover	Leguminosae	Annual
*Rumex obtusifolius* L.	Bitter dock	Polygonaceae	Perennial
*Anagallis arvensis* L.	Blue pimpernel	Primulaceae	Annual
*Chenopodium album* L.	Common goosefoot	Amaranthaceae	Annual
*Sonchus arvensis* L.	Perennial sow thistle	Asteraceae	Perennial
*Conyza stricta* Willd.	Horseweed	Asteraceae	Annual
*Convolvulus arvensis* L.	Field bindweed	Convolvulaceae	Perennial
*Medicago polymorpha* L.	Yellow trefoil	Leguminosae	Annual
*Coronopus didymus* L. Sm.	Swine-cress	Brassicaceae	Annual
Grassy weed species			
*Polypogon monspeliensis* L. Desf.	Winter grass	Poaceae	Annual
*Spergula arvensis* L.	Corn spurry	Caryophyllaceae	Annual
*Bolboschoenus maritimus* (L.) Palla	Salt marsh	Cyperaceae	Perennial

**Table 2:** Influence of different barley-based cropping systems on the overall density (m^−2^) of broadleaved and grassy weed species under various weed management methods during 2017–2018 and 2018–2019.

Cropping Systems	2017–2018					2018–2019				
	WC	FS	CC	AWE	Means (CS)	WC	FS	CC	AWE	Means (CS)
Broadleaved weeds (m^−2^)										
FB	67.00 b	30.00 f	0.33 j	39.00 d	27.27 B	84.00 b	41.33 de	0.00 k	37.67 ef	32.60 B
MB	35.67 de	18.00 h	0.00 j	16.33 h	14.00 D	33.00 gh	14.67 j	0.00 k	12.67 j	12.06 D
CB	73.00 a	34.67 e	1.33 j	47.33 c	31.27 A	67.33 c	30.00 hi	0.00 k	26.00 i	24.67 C
M*B	70.00 ab	24.00 g	0.33 j	32.33 ef	25.33 C	98.67 a	44.00 d	3.67 k	36.00 fg	36.47 A
SB	32.67 ef	9.67 i	0.00 j	11.33 i	10.73 E	29.33 hi	10.33 j	0.00 k	10.33 j	10.00 E
Means (WCS)	55.67 A	23.27 C (58.20)	0.40 D (99.28)	29.27 B (47.42)		62.47 A	28.07 B (55.06)	0.73 D (98.83)	24.53 C (60.72)	
LSD value (*p* < 0.05)	WCS = 1.51, CS = 1.51, WCS × CS = 3.38					WCS = 1.98, CS = 1.98, WCS × CS = 4.44				
Grassy weeds (m^−2^)										
FB	43.00 a	27.00 c	4.33 h–k	12.33 e	17.33 A	38.67 a	14.33 e–g	7.67 h	19.67 c	16.06 A
MB	9.33 f	2.67 j–m	0.67 m	4.00 i–l	3.33 D	16.67 de	6.67 h	3.00 ij	8.00 h	6.87 C
CB	39.00 b	18.00 d	5.67 hi	8.67 fg	14.27 B	37.67 a	12.00 g	5.67 hi	18.33 cd	14.73 B
M*B	29.00 c	8.33 fg	2.00 k–m	5.00 h–j	8.86 C	40.00 a	15.00 ef	3.33 ij	22.67 b	16.20 A
SB	6.67 gh	2.33 k–n	0.67 m	1.67 k–m	2.27 D	12.67 fg	3.00 ij	0.67 j	5.67 hi	4.40 D
Means (WCS)	25.40 A	11.67 B (54.05)	2.67 D (89.48)	6.33 C (75.07)		29.13 A	10.20 C (64.98)	4.07 D (86.02)	14.87 B (48.95)	
LSD value (*p* < 0.05)	WCS = 1.13, CS = 1.13, WCS × CS = 2.52					WCS = 1.31, CS = 1.31, WCS × CS = 2.92				

Means not having common letter for individual and interactive effects significantly vary from each other at *p* ≤ 0.05. Here, WF = weed free (control), WC = weedy check (control), FS = false seedbed, CC = chemical control, AWE = allelopathic water extracts, FB = fallow-barley, MB = maize-barley, CB = cotton-barley, M*B = mungbean-barley, SB = sorghum-barley, WCS = weed control strategies, CS = cropping system, DAS = days after sowing. The values presented in brackets indicated the % decrease in the number of broadleaf weeds than WC (control).

**Table 3:** Influence of different barley-based cropping systems on individual density (m^−2^) of grassy weed species under various weed management methods during 2017–2018 and 2018–2019.

Cropping Systems	2017–2018					2018–2019				
	WC	FS	CC	AWE	Means	WC	FS	CC	AWE	Means
Salt marsh										
FB	16.00 a	10.33 c	1.33 hi	6.00 de	6.73 A	14.00 b	4.00 g–i	2.67 ij	6.33 d–f	5.40 A
MB	4.67 ef	1.00 hi	0.00 i	1.33 hi	1.40 D	7.00 de	2.33 ij	1.33 jk	3.33 hi	2.80 C
CB	12.33 b	6.67 d	2.00 gh	3.00 g	4.80 B	11.67 c	3.33 hi	1.33 jk	5.33 e–g	4.33 B
M*B	9.33 c	3.33 fg	1.00 hi	1.00 hi	2.93 C	16.33 a	5.00 f–h	1.00 jk	8.00 d	6.07 A
SB	3.00 g	1.00 hi	0.00 i	0.67 hi	0.93 D	6.33 d–f	1.33 jk	0.00 k	2.67 ij	2.07 C
Means (WCS)	9.07 A	4.47 B (50.71)	0.87 D (90.40)	2.40 C (73.53)		11.07 A	3.20 C (71.09)	1.27 D (88.52)	5.13 B (53.65)	
LSD value (*p* < 0.05)	WCS = 0.68, CS = 0.68, WCS × CS = 1.53					WCS = 0.83, CS = 0.83, WCS × CS = 1.85				
Corn spurry										
FB	3.33 b	1.33 c	0.00 d	1.00 c	1.13 B	7.67 a	3.33 c	1.67 d	4.00 c	3.33 A
MB	0.67 cd	0.00 d	0.00 d	0.67 cd	0.27 C	1.00 d–f	1.67 d	0.33 ef	0.00 f	0.60 C
CB	6.00 a	1.33 c	0.67 cd	0.67 cd	1.73 A	6.00 b	1.33 de	1.33 de	3.33 c	2.40 B
M*B	3.00 b	0.67 cd	0.00 d	0.00 d	0.73 B	0.00 f	1.00 d–f	0.00 f	2.00 d	0.60 C
SB	0.67 cd	0.00 d	0.00 d	0.00 d	0.13 C	0.00 f	0.00 f	0.00 f	0.00 f	0.00 D
Means (WCS)	2.73 A	0.67 B (75.45)	0.13 CD (95.23)	0.47 BC (82.78)		2.93 A	1.47 B (49.82)	0.67 C (77.13)	1.87 B (36.17)	
LSD value (*p* < 0.05)	WCS = 0.44, CS = 0.44, WCS × CS = 0.99					WCS = 0.45, CS = 0.45, WCS × CS = 1.01				
Winter grass										
FB	10.67 b	5.00 d	1.00 ef	0.00 f	3.33 B	0.00	0.00	0.00	0.00	0.00
MB	0.00 f	0.00 f	0.00 f	0.00 f	0.00 D	0.00	0.00	0.00	0.00	0.00
CB	12.00 a	5.00 d	1.33 e	1.00 ef	3.87 A	0.00	0.00	0.00	0.00	0.00
M*B	9.00 c	0.00 f	0.00 f	1.33 e	2.07 C	0.00	0.00	0.00	0.00	0.00
SB	0.00 f	0.00 f	0.00 f	0.00 f	0.00 D	0.00	0.00	0.00	0.00	0.00
Means (WCS)	6.33 A	2.00 B (68.4)	0.47 C (92.57)	0.47 C (92.57)		0.00	0.00	0.00	0.00	
LSD value (*p* < 0.05)	WCS = 0.52, CS = 0.52, WCS × CS = 1.16					WCS = NS, CS = NS, WCS × CS = NS				

Means not having common letter for individual and interactive effects significantly vary from each other at *p* ≤ 0.05. Here, WF = weed free (control), WC = weedy check (control), FS = false seedbed, CC= chemical control, AWE = allelopathic water extracts, FB= fallow-barley, MB = maize-barley, CB = cotton-barley, M*B = mungbean-barley, SB = sorghum-barley, WCS = weed control strategies, CS = cropping system, DAS = days after sowing, NS = Non-significant. The values presented in brackets indicated the % decrease in the number of winter grass plants than WC (control).

**Table 4:** Influence of different barley-based cropping systems on individual density (m^−2^) of broadleaved weed species under various weed management methods during 2017–2018 and 2018–2019.

Cropping Systems	2017–2018					2018–2019				
	WC	FS	CC	AWE	MEANS	WC	FS	CC	AWE	MEANS
Common goosefoot										
FB	7.33 b	4.67 cd	0.33 h	3.00 ef	3.06 A	9.00 b	2.67 e–g	0.00 i	4.33 cd	3.20 B
MB	3.33 d–f	1.33 gh	0.00 h	0.33 h	1.00 B	4.33 cd	1.00 hi	0.00 i	2.00 f–h	1.47 D
CB	8.00 b	5.67 c	0.67 h	4.00 de	3.67 A	7.67 b	1.33 g–i	0.00 i	3.00 d–f	2.40 C
M*B	10.33 a	2.33 fg	0.00 h	3.33 d–f	3.20 A	12.67 a	3.67 de	0.00 i	5.67 c	4.40 A
SB	3.00 ef	1.00 gh	0.00 h	1.00 gh	1.00 B	4.00 de	1.00 hi	0.00 i	1.33 g–i	1.27 D
Means (WCS)	6.40 A	3.00 B (53.12)	0.20 D (96.87)	2.33 C (63.59)		7.53 A	1.93 C (74.36)	0.00 D (100)	3.27 B (56.57)	
LSD value (*p* < 0.05)	WCS = 0.66, CS = 0.66, WCS × CS = 1.48					WCS = 0.68, CS = 0.68, WCS × CS = 1.53				
Perennial sow thistle										
FB	4.33 a	2.33 b	0.00 e	1.33 b–d	1.60	7.67 b	2.67 e–h	0.00 j	3.67 c–e	2.80
MB	1.33 b–d	0.67 de	0.00 e	0.67 de	0.53	3.33 d–f	1.00 ij	0.00 j	1.67 g–i	1.20
CB	2.33 b	1.00 c–e	0.67 de	1.00 c–e	1.00	8.33 ab	2.33 e–i	0.00 j	5.00 c	3.13
M*B	5.00 a	2.00 bc	0.00 e	1.67 b–d	1.73	9.33 a	2.33 e–i	1.00 ij	4.67 cd	3.47
SB	1.67 b–d	1.00 c–e	0.00 e	0.67 de	0.67	3.00 e–g	1.33 h–j	0.00 j	2.00 f–i	1.27
Means (WCS)	2.93 A	1.40 B (52.21)	0.13 C (95.56)	1.07 B (63.48)		6.33 A	1.93 C (69.51)	0.20 D (96.84)	3.40 B (46.28)	
LSD value (*p* < 0.05)	WCS = 0.48, CS = NS, WCS × CS = 1.08					WCS = 0.68, CS = NS, WCS × CS = 1.51				
Bitter dock										
FB	17.67 b	9.00 ef	0.00 j	14.33 c	8.20 B	24.33 b	15.00 e	0.00 m	8.33 hi	9.53 A
MB	9.00 ef	3.67 hi	0.00 j	4.67 h	3.47 D	10.33 g	7.00 ij	0.00 m	3.00 kl	4.07 C
CB	20.33 a	10.67 de	0.00 j	16.33 b	9.47 A	20.00 c	12.67 f	0.00 m	6.33 j	7.80 B
M*B	13.33 c	6.67 g	0.00 j	11.00 d	6.20 C	26.33 a	17.67 d	0.00 m	7.00 ij	10.20 A
SB	6.00 fg	2.00 i	0.00 j	2.67 i	2.53 E	9.00 gh	4.33 k	0.00 m	1.67 lm	3.00 D
Means (WCS)	13.67 A	6.40 C (53.18)	0.00 D (100)	9.80 B (28.31)		18.00 A	11.33 B (37.05)	0.00 D (100)	5.27 C (70.72)	
LSD value (*p* < 0.05)	WCS = 0.84, CS = 0.84, WCS × CS = 1.88					WCS = 83, CS = 0.83, WCS × CS = 1.86				
Fat hen										
FB	6.33 a	4.00 bc	0.00 h	4.00 bc	2.87 A	6.67 a	3.00 bc	0.00 g	2.67 cd	2.47 A
MB	2.00 d–f	2.00 d–f	0.00 h	1.67 e–g	1.13 C	1.67 d–f	1.00 e–g	0.00 g	1.33 ef	0.80 CD
CB	4.00 bc	2.33 b–f	0.00 h	2.67 de	1.80 B	4.00 b	2.00 c–e	0.00 g	1.67 d–f	1.53 B
M*B	5.00 b	1.67 e–g	0.00 h	3.00 cd	1.93 B	3.00 bc	1.33 ef	0.00 g	1.00 e–g	1.07 BC
SB	2.00 d–f	1.33 fg	0.00 h	0.67 gh	0.80 C	1.33 ef	0.67 fg	0.00 g	0.67 fg	0.53 D
Means (WCS)	3.87 A	2.27 B (41.34)	0.00 C (100)	2.40 B (37.98)		3.33 A	1.60 B (51.95)	0.00 C (100)	1.47 B (55.85)	
LSD value (*p* < 0.05)	WCS = 0.56, CS = 0.56, WCS × CS = 1.24					WCS = 0.49, CS = 0.49, WCS × CS = 1.10				

Means not having common letter for individual and interactive effects significantly vary from each other at *p* ≤ 0.05. Here, WF = weed free (control), WC = weedy check (control), FS = false seedbed, CC = chemical control, AWE = allelopathic water extracts, FB = fallow-barley, MB = maize-barley, CB = cotton-barley, M*B = mungbean-barley, SB = sorghum-barley, WCS = weed control strategies, CS = cropping system, DAS = days after sowing. The values presented in brackets indicated the % decrease in the number of fat hen plants than WC (control).

**Table 5:** Influence of different barley-based cropping systems on individual weeds density (m^−2^) under various weed management methods during 2017–2018 and 2018–2019.

Cropping Systems	2017–2018					2018–2019				
	WC	FS	CC	AWE	MEANS	WC	FS	CC	AWE	MEANS
Field bindweed										
FB	0.00 f	0.00 f	0.00 f	0.00 f	0.00 ^NS^	0.00 b	0.00 b	0.00 b	0.00 b	0.00 ^NS^
MB	1.67 bc	0.67 d–f	0.00 f	1.00 c–e	0.67	0.00 b	0.00 b	0.00 b	0.00 b	0
CB	2.00 b	0.67 d–f	0.00 f	0.33 ef	0.6	0.00 b	0.00 b	0.00 b	0.00 b	0
M*B	4.00 a	1.67 bc	0.33 ef	1.33 b–d	1.46	0.00 b	1.33 a	0.00 b	0.00 b	0.27
SB	1.33 b–d	0.00 f	0.00 f	0.67 d–f	0.4	0.00 b	0.00 b	0.00 b	0.00 b	0
Means (WCS)	1.80 A	0.60 B (66.67)	0.07 C (96.11)	0.67 B (62.77)		0.00 B	0.27 A (100)	0.00 B (100)	0.00 B (100)	
LSD value (*p* < 0.05)	WCS = 0.38, CS = NS, WCS × CS = 0.85					WCS = 0.08, CS = NS, WCS × CS = 0.19				
Yellow trefoil										
FB	13.00 b	7.33 de	0.00 i	7.67 d	5.60 A	13.67 a	6.67 cd	0.00 i	8.67 b	5.80 A
MB	10.33 c	5.00 ef	0.00 i	2.33 gh	3.53 B	5.00 de	1.00 hi	0.00 i	2.00 f–h	1.60 C
CB	12.67 b	7.00 d	0.00 i	5.00 ef	4.93 A	9.33 b	3.33 ef	0.00 i	5.33 d	3.60 B
M*B	18.33 a	6.33 de	0.00 i	4.00 fg	5.73 A	15.33 a	6.00 d	1.33 g–i	8.33 bc	6.20 A
SB	8.00 d	3.00 gh	0.00 i	1.33 hi	2.47 C	5.67 d	1.33 g–i	0.00 i	3.00 fg	2.00 C
Means (WCS)	12.47 A	5.73 B (54.05)	0.00 D (100)	4.07 C (67.36)		9.80 A	3.67 C (62.55)	0.27 D (97.24)	5.47 B (44.18)	
LSD value (*p* < 0.05)	WCS = 0.89, CS = 0.89, WCS × CS = 2.00						WCS = 0.78, CS = 0.78, WCS × CS = 1.75			
Yellow sweet clover										
FB	14.33 c	2.67 ij	0.00 k	7.33 ef	4.87 B	16.00 b	9.00 d	0.00 l	6.67 ef	6.33 A
MB	8.00 e	4.67 g–i	0.00 k	5.33 f–h	3.60 C	6.33 e–g	3.00 i–k	0.00 l	1.67 j–l	2.20 C
CB	20.33 a	7.33 ef	0.00 k	16.67 b	8.87 A	12.33 c	5.67 fg	0.00 l	3.33 h–j	4.27 B
M*B	12.00 d	3.33 h–j	0.00 k	8.00 e	4.67 B	18.33 a	7.67 de	1.33 kl	4.67 g–i	6.40 A
SB	6.67 e–g	1.33 jk	0.00 k	4.00 hi	2.40 D	5.00 f–h	1.67 j–l	0.00 l	1.67 j–l	1.67 C
Means (WCS)	12.27 A	3.87 C (68.45)	0.00 D (100)	8.27 B (32.59)		11.60 A	5.40 B (53.44)	0.27 D (97.67)	3.60 C (68.96)	
LSD value (*p* < 0.05)	WCS = 0.90, CS = 0.90, WCS × CS = 2.01					WCS = 0.82, CS = 0.82, WCS × CS = 1.83				
Swine cress										
FB	4.00 a	0.00 d	0.00 d	1.33 c	1.06 ^NS^	2.33 c	1.00 ef	0.00 g	1.33 de	0.93 B
MB	0.00 d	0.00 d	0.00 d	0.33 d	0.06	1.00 ef	0.67 e–g	0.00 g	0.33 fg	0.40 C
CB	2.00 b	0.00 d	0.00 d	1.33 c	0.67	3.67 b	2.00 cd	0.00 g	1.33 de	1.40 A
M*B	2.00 b	0.00 d	0.00 d	0.00 d	0.4	5.33 a	2.33 c	0.00 g	1.00 ef	1.73 A
SB	0.00 d	0.00 d	0.00 d	0.00 d	0	0.67 e–g	0.00 g	0.00 g	0.00 g	0.13 C
Means (WCS)	1.60 A	0.00 C (100)	0.00 C (100)	0.60 B (62.5)		2.60 A	1.20 B (53.84)	0.00 D (100)	0.80 C (69.23)	
LSD value (*p* < 0.05)	WCS = 0.29, CS = 0.29, WCS × CS = 0.65					WCS = 0.39, CS = 0.39, WCS × CS = 0.88				

Means not having common letter for individual and interactive effects significantly vary from each other at *p* ≤ 0.05. Here, WF = weed free (control), WC = weedy check (control), FS = false seedbed, CC = chemical control, AWE = allelopathic water extracts, FB = fallow-barley, MB = maize-barley, CB = cotton-barley, M*B = mungbean-barley, SB = sorghum-barley, WCS = weed control strategies, CS = cropping system, DAS = days after sowing, NS = Non-significant. The values presented in brackets indicated the % decrease in the number of winter grass plants than WC (control).

**Table 6:** Influence of different barley-based cropping systems on individual weeds density (m^−2^) under various weed management methods during 2017–2018 and 2018–2019.

Cropping Systems	2017–2018					2018–2019				
	WC	FS	CC	AWE	MEANS	WC	FS	CC	AWE	MEAN
Blue pimpernel										
FB	0.00	0.00	0.00	0.00	0.00	3.00 b	1.33 de	0.00 f	2.00 cd	1.27
MB	0.00	0.00	0.00	0.00	0.00	1.00 e	0.00 f	0.00 f	0.67 ef	0.33
CB	0.00	0.00	0.00	0.00	0.00	0.00 f	0.00 f	0.00 f	0.00 f	0.00
M*B	0.00	0.00	0.00	0.00	0.00	4.33 a	1.33 de	0.00 f	2.33 bc	1.60
SB	0.00	0.00	0.00	0.00	0.00	0.67 ef	0.00 f	0.00 f	0.00 f	0.13
Means (WCS)	0.00	0.00	0.00	0.00		1.80 A	0.53 C (70.55)	0.00 D (100)	1.00 B (44.44)	
LSD value (*p* < 0.05)	WCS = NS, CS = NS, WCS × CS = NS					WCS = 0.39, CS = NS, WCS × CS = 0.88				
Horseweed										
FB	0.00	0.00	0.00	0.00	0.00	1.33 c	0.00 d	0.00 d	0.00 d	0.27
MB	0.00	0.00	0.00	0.00	0.00	0.00 d	0.00 d	0.00 d	0.00 d	0.00
CB	0.00	0.00	0.00	0.00	0.00	2.00 b	0.00 d	0.00 d	0.00 d	0.40
M*B	0.00	0.00	0.00	0.00	0.00	2.67 a	0.00 d	0.00 d	1.33 c	0.80
SB	0.00	0.00	0.00	0.00	0.00	0.00 d	0.00 d	0.00 d	0.00 d	0.00
Means (WCS)	0.00	0.00	0.00	0.00		1.20 A	0.00 C	0.00 C	0.27 B	
LSD value (*p* < 0.05)	WCS = NS, CS = NS, WCS × CS = NS					WCS = 0.25, CS = NS, WCS × CS = 0.56				

Means not having common letter for individual and interactive effects significantly vary from each other at *p* ≤ 0.05. Here, WF = weed free (control), WC = weedy check (control), FS = false seedbed, CC = chemical control, AWE = allelopathic water extracts, FB = fallow-barley, MB = maize-barley, CB = cotton-barley, M*B = mungbean-barley, SB = sorghum-barley, WCS = weed control strategies, CS = cropping system, DAS = days after sowing, NS = Non-significant. The values presented in brackets indicated the % decrease in the number of winter grass plants than WC (control).

**Table 7:** Influence of different barley-based cropping systems on dry biomass yield (g m^−2^) under various weed management methods.

Cropping Systems	2017–2018					
	WF	WC	FS	CC	AWE	Means (CS)
FB	332.99	248.14	316.97	330.07	324.63	310.56 C
MB	349.35	273.39	329.00	343.35	336.53	326.32 AB
CB	345.79	265.06	328.92	341.49	332.83	322.82 B
M*B	369.85	259.24	342.94	346.29	341.36	331.94 A
SB	338.56	258.07	328.65	337.08	327.66	318.00 BC
Means (WCS)	347.31 A	260.78 D	329.30 C	339.65 AB	332.60 BC	
LSD at *p* ≤ 0.05	WCS = 9.03, CS = 9.03, WCS × CS = NS					
	2018–2019					
FB	334.33	256.28	316.50	330.40	322.85	312.07 C
MB	346.86	277.38	325.03	343.37	331.20	324.77 B
CB	351.25	270.65	326.08	339.24	328.52	323.15 B
M*B	372.25	267.95	343.48	346.43	340.27	334.07 A
SB	340.15	266.45	326.05	336.65	326.11	319.08 BC
Means (WCS)	348.97 A	267.74 D	327.43 C	339.22 B	329.79 C	
LSD at *p* ≤ 0.05	WCS = 8.90, CS = 8.90, WCS × CS = NS					

Means not having common letter for individual and interactive effects significantly vary from each other at *p* ≤ 0.05. Here, WF = weed free (control), WC = weedy check (control), FS = false seedbed, CC = chemical control, AWE = allelopathic water extracts, FB = fallow-barley, MB = maize-barley, CB = cotton-barley, M*B = mungbean-barley, SB = sorghum-barley, WCS = weed control strategies, CS = cropping system, DAS = days after sowing, NS = Non-significant.

**Table 8:** Weather data for the period of research at the experimental site.

Months	2017–2018				2018–2019			
	Mean Temperature (°C)	Mean Relative Humidity (%)	Mean Daily Sunshine (h)	Total Monthly Rainfall (mm)	Mean Temperature (°C)	Mean Relative Humidity (%)	Mean Daily Sunshine (h)	Total Monthly Rainfall (mm)
May	34.00	63.05	4.80	0.10	32.90	52.60	10.30	0.00
June	33.10	74.90	4.50	45.60	34.60	64.70	3.50	0.00
July	33.65	73.00	7.20	4.90	33.20	71.20	5.50	0.00
August	31.80	85.20	7.70	30.00	32.40	75.10	4.30	0.00
September	30.60	77.10	8.00	10.00	29.80	77.10	6.80	0.00
October	27.00	77.60	7.40	4.20	23.00	75.10	5.50	0.00
November	18.00	81.40	3.70	16.00	18.90	82.25	4.40	0.00
December	14.65	75.00	5.20	16.00	14.25	85.00	5.90	0.00
January	13.65	83.10	4.40	0.00	12.20	86.35	4.30	11.00
February	17.50	75.40	4.90	6.80	14.45	80.60	6.70	25.10
March	23.50	70.90	7.20	0.00	19.55	75.95	7.30	21.00
April	29.45	56.70	5.40	3.00	28.60	73.15	7.70	12.70

**Table 9:** Crop husbandry of different crops included in barley-based cropping systems of the study.

Crops	Sowing Time	Cultivars	Seed Rate (kg ha^−1^)	Fertilizer NPK (kg ha^−1^)	P–P (cm)	R–R (cm)	Harvest Date
Year 2017 and 2018 (Summer Season)							
Cotton	15 May	IUB-2013	25	250–200–0	20	75	28 October
Sorghum	10 June	YS-16	10	100–60–0	15	60	29 October
Mungbean	15 June	NIAB-Mung 2011	20	20–60–0	10	30	27 September
Maize	25 July	YH-1898	25	200–150–0	22	75	30 October
Year 2017–2018 and 2018–2019 (Winter Season)							
Barley	10 November	Haider-93	80	50–25–0		25	7 and 10 April

P–P = Plant spacing; R–R = Row spacing.

## Data Availability

All data are within the manuscript.
